# Basal cell adenocarcinoma of the parotid gland: prognostic factors and survival outcomes: a systematic review and pooled patient-level analysis

**DOI:** 10.1007/s00405-026-10313-w

**Published:** 2026-05-21

**Authors:** Francesco Chiari, Cecilia Dalmazzini, Matteo Fermi, Livio Presutti, Claudio Donadio Caporale, Pierre Guarino

**Affiliations:** 1 Otolaryngology Head and Neck Unit - “Santo Spirito” Hospital, Pescara, Italy; 2 Department of Otolaryngology - Head and Neck Surgery, IRCCS Azienda Ospedaliero-Universitaria Di Bologna, Viale Albertoni 15, 40137 Bologna, Italy; 3 Alma Mater Studiorum, Università Di Bologna, Bologna, Italy; 4 Department of Innovative Technologies in Medicine & Dentistry, University “G. d’Annunzio” Chieti-Pescara, Chieti, Italy

**Keywords:** Parotid gland, Basal cell adenocarcinoma, Salivary gland neoplasms, Prognosis, Radiotherapy, Head and Neck

## Abstract

**Background:**

Basal cell adenocarcinoma (BCAC) of the parotid gland is a rare low-grade malignancy with generally favorable prognosis but potential for late recurrence. This systematic review aimed to evaluate survival outcomes and prognostic factors in patients with parotid BCAC.

**Methods:**

A PRISMA-compliant systematic review was conducted using Embase, PubMed, Scopus, and Cochrane Library (1990–2025). Case reports and case series providing individual patient data were included. A pooled patient-level analysis assessed local, regional, and distant disease control, as well as disease-free (DFS), disease-specific (DSS), and overall survival (OS).

**Results:**

Twenty-three studies including 68 patients were analyzed. The mean age at diagnosis was 58.6 years. The 5-year DFS, DSS, and OS were 84.1%, 92.7%, and 88.3%, respectively. Overall recurrence occurred in 31% of cases, mainly at the primary site. The absence of adjuvant therapy and perineural invasion were significantly associated with reduced DFS. Positive surgical margins were associated with worse DSS, while facial nerve palsy at presentation associated with decreased OS. Advanced tumor stage showed a trend toward poorer outcomes. Late recurrences and distant metastases have been reported in the literature beyond 5 years of follow-up.

**Conclusions:**

Parotid BCAC generally demonstrates favorable survival; however, adverse pathological features negatively impact prognosis. Adjuvant radiotherapy may improve outcomes in high-risk patients. Prolonged follow-up beyond the conventional 5-year period may be considered, particularly in high-risk patients.

## Introduction

Basal cell adenocarcinoma (BCAC) is a low-grade malignant epithelial tumor that arises predominantly in the parotid gland accounting for less than 2% of all salivary gland malignancies. From a pathological perspective, BCAC can arise de novo or, less commonly, in association with a pre-existing basal cell adenoma (carcinoma ex basal cell adenoma), a distinction that may influence tumor behavior and recurrence patterns. Accurate differentiation between these entities is clinically relevant, as the latter may exhibit more aggressive features than conventional BCAC [1–3].

Histologically, BCAC is characterized by basaloid cells arranged in solid, trabecular, tubular, or membranous patterns, with peripheral palisading and a lack of myxochondroid stroma, distinguishing it from pleomorphic adenoma and other basaloid neoplasms [4, 5]. Despite its generally indolent clinical behavior, BCAC has been associated in some cases with locally aggressive features, such as perineural invasion (PNI), recurrence, and rarely, regional or distant metastasis [6–8]. Cases of bilateral, synchronous, or multifocal involvement of the parotid gland have also been reported, further complicating diagnosis and management [9, 10].

A diagnostic challenge lies in the preoperative differentiation of BCAC from its benign counterpart, basal cell adenoma (BCA). Both tumors share similar cytologic and radiologic features, which can hinder accurate diagnosis prior to surgical excision. Several studies have examined imaging and cytologic criteria, but findings often remain inconclusive [11–15]. Fine needle aspiration cytology (FNAC), while useful, may not always reliably distinguish BCAC from benign lesions, underscoring the importance of histopathological and immunohistochemical evaluation [10, 16].

The mainstay of treatment for BCAC is surgical resection, most commonly in the form of superficial or total parotidectomy, depending on tumor size, location, and proximity to the facial nerve [5, 17]. In most reported cases, complete excision is associated with favorable local control. For tumors with aggressive features, such as PNI, lymphovascular invasion (LVI), close/positive surgical margins (R +), adjuvant radiotherapy (RT) may be considered, although its role is not clearly defined [18].

The current evidence remains fragmented and is largely limited to isolated case reports and small case series, resulting in heterogeneous reporting of oncologic outcomes. Moreover, long-term recurrence patterns and survival determinants are not clearly established, particularly beyond the conventional 5-year follow-up period. To address these gaps, a pooled patient-level analysis is warranted to better define prognostic factors and long-term survival outcomes in patients affected by parotid BCAC.

## Material and methods

### Search strategy and information sources

Following the PRISMA 2020 guidelines for systematic reviews and meta-analyses, a comprehensive literature review was conducted using the PICOS framework to define the search strategy and inclusion criteria [19, 20]. A computerized search was performed across the Embase, PubMed, Scopus, and Cochrane Library databases to identify articles published between 1 st January 1990 until 31 st December 2025. The search strategy combined controlled vocabulary (MeSH/Emtree terms) and free-text terms related to basal cell adenocarcinoma and the parotid gland. The core search string for PubMed was as follows: ("Basal Cell Adenocarcinoma"[Mesh] OR "basal cell adenocarcinoma" OR "BCAC") AND ("Parotid Gland"[Mesh] OR "parotid gland" OR "parotid") AND ("Salivary Gland Neoplasms"[Mesh] OR "salivary gland tumor*" OR "salivary gland neoplasm*") NOT ("skin" OR "cutaneous" OR "basal cell carcinoma"). Equivalent adaptations were applied for the other databases. A manual search of the reference lists of eligible articles was also performed to identify additional relevant studies.

### Study selection and data extraction

After executing the search strategy, the titles and abstracts retrieved were independently screened by two authors (F.C. and C.D.). Discrepancies regarding citation inclusion were resolved through discussion and consensus. The eligibility of studies was assessed based on the PICOTS criteria, as:

- Participants (patients affected by parotid BCAC);
- Intervention (patients treated with surgery with or without adjuvant medical treatments);
- Comparator (not applicable);
- Outcomes (oncological outcomes as local control of disease (LCD), regional control of disease (RCD), distant control of disease (DCD), disease free survival (DFS), disease specific survival (DSS), and overall survival (OS), and prognostic factors related to survival rates);
- Time (articles published until 31 st December 2025);
- Study design (case series, case reports).

**Fig. 1:**
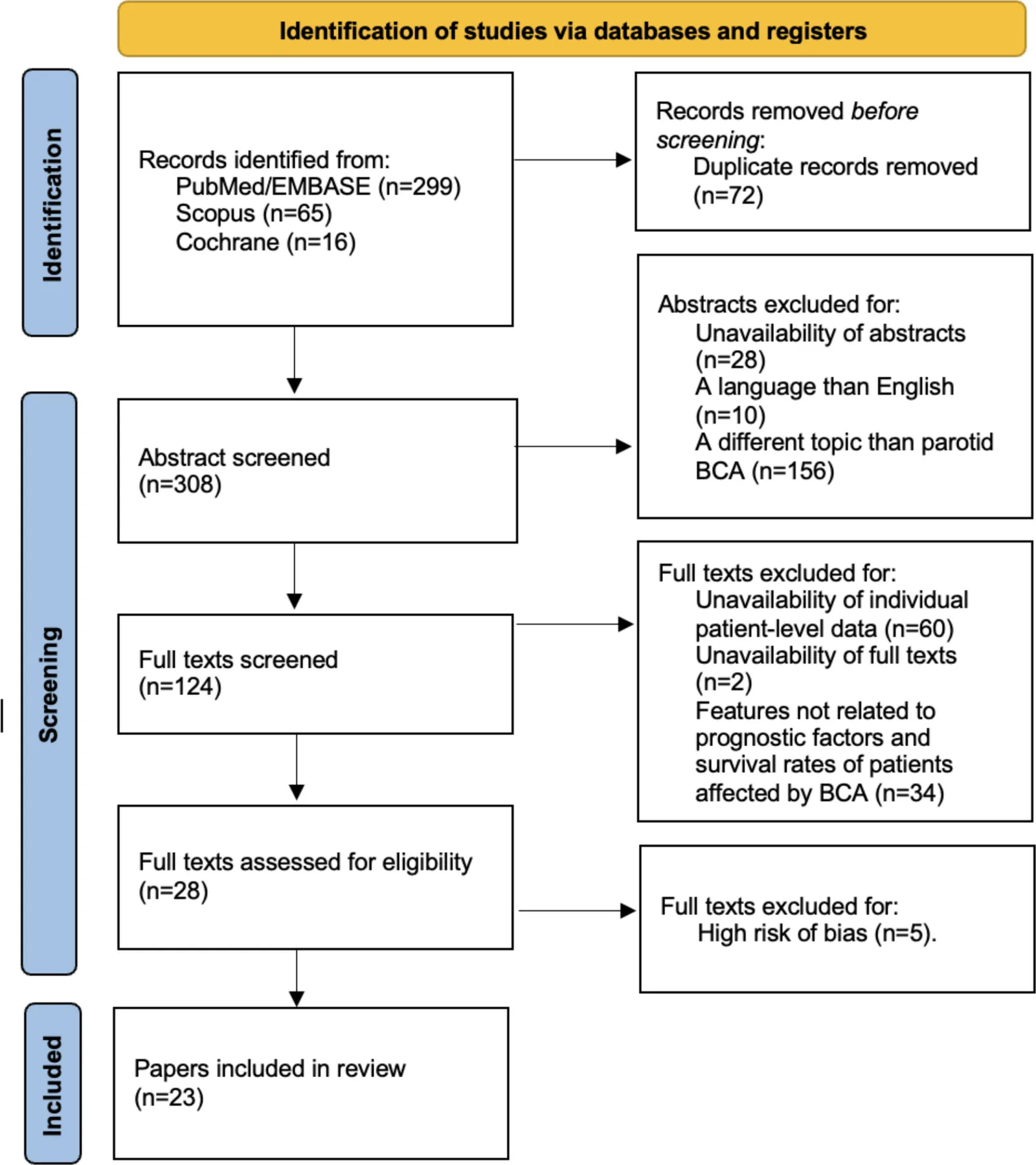
Flow chart of the study

Inclusion criteria comprised articles written in English that reported data of patients affected by BCAC. Studies were excluded if they lacked an abstract or full text, were not in English, or did not provide relevant data regarding patients affected by BCAC. A manual search of the reference lists of included studies was also performed to identify any additional relevant publications. Data from each study were extracted using a standardized data collection form. The study selection process is illustrated in the PRISMA flow diagram (Fig. 1).

The systematic review protocol has been registered in the PROSPERO database (registration number 1132439).

### Quality assessment

**Fig. 2:**
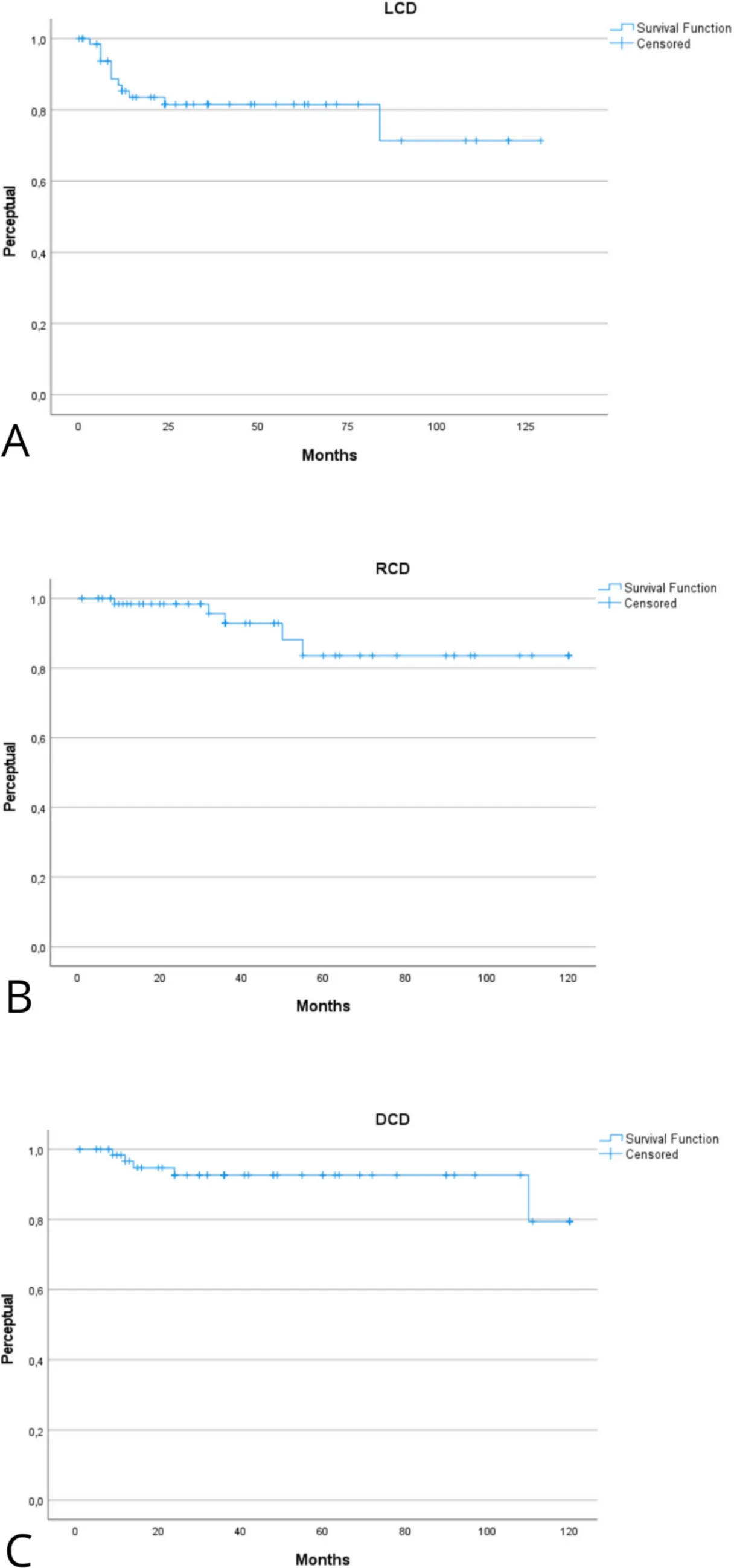
Kaplan–Meier curves showing control of disease rates: (**A**) LCD; (**B**) RCD; (**C**) DCD

**Fig. 3:**
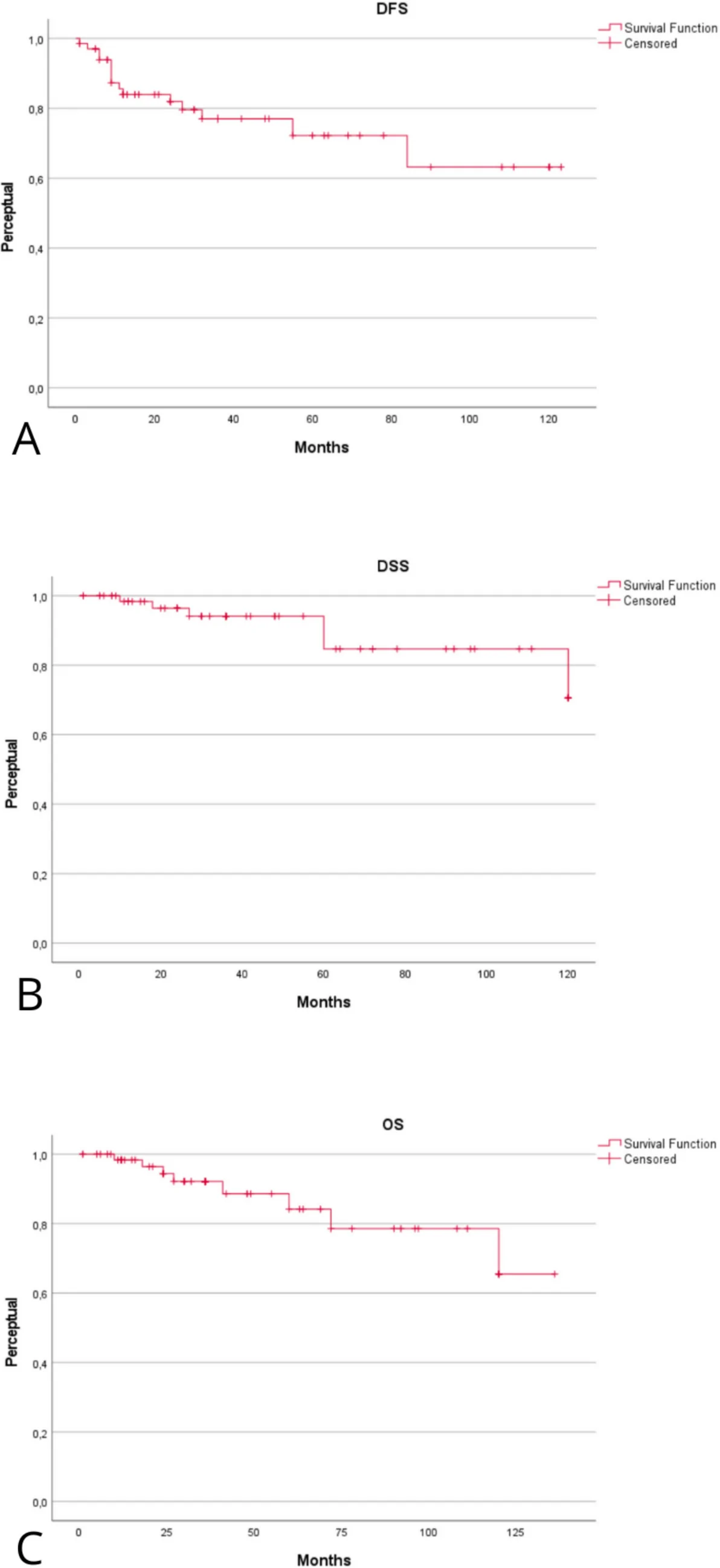
Kaplan–Meier curves showing survival rates: (**A**) DFS; (**B**) DSS; (**C**) OS

The same two authors (F.C. and C.D.) independently assessed the quality of the included studies using a standardized tool for evaluating case series and case reports [21]. This tool evaluates four domains: selection, ascertainment, causality, and reporting, through eight specific questions that contribute to an overall quality score. Based on this assessment, studies were categorized as having a low, moderate, or high risk of bias (Table 1). Articles identified as having a high risk of bias were excluded from the review. To further validate the inclusion of studies with low or moderate risk of bias, the Joanna Briggs Institute Critical Appraisal Tools was applied [22], ensuring the methodological robustness and reliability of the selected studies. Figs. 2 and 3

**Table 1:** Tool for evaluating the methodological quality of case reports and case series. The eight questions included in the evaluation tool are categorized into the following four domains: selection, ascertainment, causality and reporting. Within each domain the Authors evaluated whether the question related conditions were satisfied (1) or not (0). Final results differentiated case reports or series into 3 categories of risk of bias as follows: low risk (1) if all questions of the four domains were matched; intermediate risk (2) if questions of three out of the four domains were matched; high risk (3) if questions of less than three out of the four domains were matched

Authors	Year	Selection	Ascertainment	Causality	Reporting	Risk of bias
Terada [11]	2021	1	1	1	1	1
Vitorino [24]	2022	1	1	1	1	1
Muller [4]	1996	1	1	1	1	1
Qiao [13]	2010	1	1	0	0	3
Ülkü [6]	2017	1	1	1	1	1
Kusafuka [14]	2017	1	1	1	1	1
Nagao [15]	1997	1	1	1	1	1
Razali [7]	2019	1	1	1	0	2
Chiu [12]	2007	1	1	0	0	3
Manor [25]	2006	1	1	1	0	2
Eroglu [8]	2015	1	1	1	1	1
Moroz [16]	1996	1	1	0	0	3
Dou [1]	2024	1	1	0	0	3
Ozgun [26]	2014	1	1	1	0	2
Takeuchi [9]	2001	1	1	1	1	1
Ikeda [27]	1998	1	1	1	1	1
Ellis [18]	1990	1	1	1	0	2
Saharan [28]	2024	1	1	1	0	2
Murty [2]	1990	1	1	0	0	3
Mantsopoulos [5]	2023	1	1	1	1	1
Lin [29]	2004	1	1	1	0	2
Kim [30]	1997	1	1	1	0	2
Pisharodi [23]	1995	1	1	1	1	1
Saluja [31]	2016	1	1	1	1	1
Markkanen-Leppänen [32]	2010	1	1	1	0	2
Tse [3]	2001	1	1	1	0	2
Chung [10]	2007	1	1	1	1	1
Park [17]	2024	1	1	1	1	1

### Data analysis

A patient-level analysis was performed by extracting individual data from the included studies. Statistical analyses were conducted using IBM SPSS Statistics version 27.0. Continuous variables were reported as mean ± standard deviation (SD) or median and interquartile range (IQR), according to data distribution assessed using the Shapiro–Wilk test. Categorical variables were compared using the Chi-square test or Fisher's exact test, as appropriate. Survival outcomes (DFS, DSS, OS, LCD, RCD, and DCD) were estimated using the Kaplan–Meier method, and differences between groups were assessed using the log-rank test. Prognostic factors were evaluated using univariate logistic regression models, and results were expressed as odds ratios (ORs) with 95% confidence intervals (CI). Regression was performed on binary endpoints (event vs no event). Given the limited sample size and heterogeneity of follow-up duration among included studies, regression analyses were performed based on the occurrence of events rather than time-dependent modelling. Multivariable analysis was not performed due to the small number of events. Statistical significance was defined as a two-sided p-value < 0.05. Endpoints were defined as follows:

- LCD: absence of recurrence at the primary tumor site;
- RCD: absence of cervical lymph node recurrence;
- DCD: absence of distant metastasis;
- DFS: time from treatment to recurrence or last follow-up;
- DSS: time from treatment to disease-related death;
- OS: time from treatment to death from any cause.

Percentages were calculated based on the number of evaluable cases for each variable. No imputation for missing data was performed.

## Results

A total of 299, 65, and 16 records were identified through Embase/PubMed, Scopus, and Cochrane databases, respectively. After removal of duplicates, 308 unique records were screened. Following title and abstract evaluation, 124 articles underwent full-text assessment. Of these, 101 studies were excluded for the following reasons: lack of individual patient-level data (n = 60), irrelevance to prognostic factors or survival outcomes (n = 34), and full-text unavailability (n = 7). After quality assessment, 5 additional studies were excluded due to high risk of bias, as summarized in Table 1. In total, 23 studies were included in the final qualitative and quantitative analysis, comprising 6 case series [4, 5, 11, 15, 17, 18] and 17 case reports [3, 6–10, 14, 23–32], published between 1990 and 2025. The largest cohort was reported by Ellis et al. [18], including 24 patients. The study selection process is reported in Fig. 1.

### Cohort characteristics

Table 2 describes the main cohort’s features. A total of 68 patients were included in the final analysis, comprising 25 males (37%) and 43 females (63%). The mean age at diagnosis was 58.6 ± 12.9 years (median 57 years, IQR 49–67). Preoperative imaging included computed tomography in 70% of patients, ultrasonography in 50%, and magnetic resonance imaging in 30%. FNAC was performed in 31% of cases, with cytological results suggestive of malignancy in 57%, benign neoplasms in 29%, and non-diagnostic in 14%. The mean tumor diameter was 32.1 ± 13.1 mm (median 30 mm, IQR 22–40). Clinical examination revealed pre-treatment facial nerve palsy in 8% of patients. According to the AJCC 8th edition staging system, 66% of patients presented with early-stage disease (stage I–II), while clinical nodal metastases were identified in 6%. Pathological staging revealed a higher proportion of advanced-stage tumors (58%) compared with clinical staging, reflecting discrepancies between preoperative and postoperative assessment and suggesting a relevant rate of pathological upstaging. Surgical treatment of the primary tumor included total parotidectomy in 36%, subtotal parotidectomy in 23%, superficial parotidectomy in 23%, and parotid node resection in 18% of patients. Among the studies included in the systematic review, the European Salivary Gland Society classification system was not applied, as it was reported in only a very limited number of articles. In addition, selective neck dissection (SND) was performed in 25% of cases. However, the indication of SND was not consistently specified across studies. Histopathological evaluation revealed PNI, LVI and R + in 40%, 17%, and 27% cases, respectively. Despite the relatively high proportion of tumors with advanced pathological stage or adverse features, adjuvant therapies were administered in 35% of patients, consisting of RT in 84% and chemotherapy (CHT) in 16%. The indication for adjuvant treatment was not consistently reported across studies, and treatment decisions appeared heterogeneous. Tumor grading was inconsistently reported across the included studies. In the majority of cases, BCAC was described as a low-grade malignancy, and no cases of high-grade transformation were explicitly documented in the available data. Genetic and syndromic data were not consistently reported across the included studies.

**Table 2:** Cohort characteristics

Variable		Nr. Cases
Patient features	Total patients included Males: cases (%) Females: cases (%) Mean age: years (range)	68 25 (37%) 43 (63%) 58.60 (27—92)
Risk factors	Smoke: cases (%) Alcohol: cases (%) Diabetes mellitus: cases (%) Immunodeficiency: cases (%) Previous parotid tumors: cases (%)	4 (40%) 2 (20%) 2 (20%) 1 (10%) 1 (10%)
Diagnostic tools	CT: cases (%) US: cases (%) MRI cases (%) PET: cases (%) FNA: cases (%) - malignant diagnosis: cases (%) - benign diagnosis: cases (%) - not diriment: cases (%)	7 (70%) 5 (50%) 3 (30%) 1 (10%) 21 (31%) 12 (57%) 6 (29%) 3 (14%)
Clinical features	Maximum diameter of T: mm (range) Facial palsy: cases (%) cT early: cases (%) cT advanced: cases (%) cN metastasis: cases (%)	32.07 (17—60) 5 (8%) 35 (66%) 18 (34%) 3 (6%)
Treatment	Parotid node resection: cases (%) Superficial parotidectomy: cases (%) Sub-total parotidectomy: cases (%) Total parotidectomy: cases (%) SND: cases (%) Adjuvant therapy: cases (%) - RT: cases (%) - CHT: cases (%)	6 (18%) 8 (23%) 8 (23%) 12 (36%) 10 (25%) 13 (35%) 11 (84%) 2 (16%)
Pathological features	PNI + : cases (%) LVI + : cases (%) R + : cases (%) pT early: cases (%) pT advanced: cases (%) pN metastasis: cases (%)	10 (40%) 5 (17%) 3 (27%) 15 (42%) 21 (58%) 2 (6%)
Recurrences	Local recurrences: cases (%) Regional recurrences: cases (%) Distant recurrences: cases (%)	12 (18%) 4 (6%) 5 (7%)
Follow-up	Alive without disease: cases (%) Death for disease: cases (%) Mean follow up: months (range)	57 (84%) 5 (7%) 46.40 (1—201)

Abbreviations: CHT = chemotherapy; CT = computed tomography; DFS = disease free survival; DSS = disease specific survival; FNA = fine needle aspiration; mm = millimetres; MRI = magnetic resonance imaging; N = lymph node; OS = overall survival; PET = positron emission tomography; R = surgical margin; RT = radiotherapy; SND = selective node dissection; T = tumor; US = ultrasonography

Addendum: percentages were calculated on the number of patients with available data for each variable (denominator varies)

### Recurrence patterns, survival outcomes, and prognostic factors

Table 2 defines the patterns of disease recurrence and key survival outcomes. The mean follow-up duration was 46.4 ± 40.7 months (median 36 months, IQR 18–60), ranging from 1 to 201 months. At the time of last follow-up, 84% of patients were alive without evidence of disease, while 7% had died due to disease progression. Overall recurrence occurred in 31% of cases, predominantly at the primary site (18%), followed by regional lymph nodes (6%) and distant sites (7%). However, this recurrence rate should be interpreted cautiously, as the pooled cohort included patients treated over a long-time span with heterogeneous and often incompletely reported diagnostic workup and treatment strategies. Therefore, part of the observed recurrence burden may reflect differences in management rather than the intrinsic biological aggressiveness of the disease alone.

Table 3 summarizes univariate analyses for disease control outcomes. The absence of adjuvant therapy was significantly associated with reduced LCD (OR 1.44; 95% CI 1.14–2.93; p = 0.007), as was the presence of PNI (OR 1.42; 95% CI 1.15–1.89; p = 0.024). R + showed a trend toward worse LCD (OR 1.50; 95% CI 0.67–3.33; p = 0.087), while LVI demonstrated a non-significant association (OR 4.89; 95% CI 0.56–42.30; p = 0.125). Clinical nodal metastasis at presentation was significantly associated with reduced RCD (OR 12.00; 95% CI 7.40–194.63; p = 0.033). No variables were significantly associated with DCD.

**Table 3:** Univariate Analyses of LCD, RCD and DCD

	LCD		RCD		DCD	
*Variable*	*OR (95% CI)*	*P value*	*OR (95% CI)*	*P value*	*OR (95% CI)*	*P value*
Female gender	3.48 (0.70—17.41)	.187	0.56 (0.07—4.25)	.571	2.46 (0.26—23.34)	.419
Age > 70 years	1.74 (0.39—7.71)	.432	5.40 (0.68—42.91)	.807	1.18 (0.12—11.62)	.886
Smoke	0.44 (0.32—1.71)	.892	0.73 (0.20—3.40)	.672	0.87 (0.28—9.80)	.870
Alcohol	0.74 (0.11—3.45)	.911	0.94 (0.31—6.45)	.578	0.31 (0.11—3.44)	.778
Diabetes mellitus	0.63 (0.40—2.99)	.305	10.00 (0.31—315.27)	.140	0.91 (0.75—1.10)	.658
Immunodeficiency	0.60 (0.44—2.79)	.488	0.83 (0.64—1.07)	.657	0.92 (0.77—1.09)	.764
Facial palsy	1.27 (0.13—12.63)	.835	0.93 (0.87—2.43)	.555	4.83 (0.40—57.70)	.174
Diameter of T > 25 mm	1.25 (0.17—9.02)	.825	1.07 (0.94—1.23)	.362	0.78 (0.44—14.03)	.869
Advanced cT stage	2.22 (0.48—10.15)	.421	0.97 (0.08—11.48)	.981	0.97 (0.8—11.48)	.981
cN metastasis	1.84 (0.07—1.94)	.452	12.00 (7.40—194.63)	.033	0.94 (0.87—1.01)	.662
Adjuvant therapy	1.44 (1.14—2.93)	.007	–	1.000	4.81 (0.34—51.23)	.233
PNI +	1.42 (1.15—1.89)	.024	1.11 (0.94—1.36)	.211	1.11 (0.90—1.36)	.211
LVI +	4.89 (0.56—42.30)	.125	0.96 (0.88—1.04)	.650	6.00 (0.31—116.60)	.190
R +	1.50 (0.67—3.33)	.087	–	1.000	–	1.000
Advanced pT stage	3.33 (0.33—32.94)	.290	1.05 (0.95—1.56)	.391	0.70 (0.04—12.16)	.806
pN metastasis	0.93 (0.84—1.07)	.706	–	1.000	0.97 (0.90—1.03)	.793

Abbrev: CI, confidential interval; DC = distant control; LC = local control; LVI = lymph vascular invasion; N = lymph node; PNI = peri neural invasion; R = surgical margins; RC = regional control; T = primary tumor

Table 4 reports univariate analyses for survival outcomes. The absence of adjuvant therapy was significantly associated with DFS (OR 14.37; 95% CI 1.42–142.35; p = 0.007), DSS (OR 1.30; 95% CI 1.06–2.71; p = 0.014), and OS (OR 1.44; 95% CI 1.02–2.07; p = 0.004). The presence of PNI was associated with statistically significant reduced DFS (OR 1.66; 95% CI 1.05–2.76; p = 0.008), with a trend toward worse DSS (OR 1.21; 95% CI 0.84–1.98; p = 0.078) and OS (OR 1.25; 95% CI 0.92–1.70; p = 0.071). R + was significantly associated with decreased DSS (OR 1.52; 95% CI 1.29–1.85; p = 0.038), while showing a non-significant trend for DFS and OS (p = 0.087 for both). Facial nerve palsy at presentation was significantly associated with reduced OS (OR 7.46; 95% CI 1.01–55.70; p = 0.026). Other variables, including age > 70 years, advanced T stage, LVI, and nodal metastasis, did not reach statistical significance in survival analyses.

**Table 4:** Univariate Analyses of DFS, DSS and OS

	DFS		DSS		OS	
*Variable*	*OR (95% CI)*	*P value*	*OR (95% CI)*	*P value*	*OR (95% CI)*	*P value*
Female gender	1.08 (0.50—6.43)	.545	1.10 (0.84—1.82)	.148	1.86 (0.35—10.03)	.700
Age > 70 years	3.29 (0.86—12.49)	.119	2.03 (0.88—2.81)	.362	1.66 (0.20—9.48)	.624
Smoke	0.86 (0.48—4.33)	.578	0.36 (0.12—3.93)	.778	0.96 (0.52—3.56)	.912
Alcohol	0.80 (0.64—3.22)	.567	0.40 (0.11—4.55)	.611	0.70 (0.31—2.52)	.814
Diabetes mellitus	1.75 (0.08—36.29)	.715	4.30 (0.31—13.72)	.087	0.81 (0.62—1.08)	.512
Immunodeficiency	0.58 (0.36—2.94)	.411	0.97 (0.64—3.07)	.555	0.83 (0.64—1.07)	.657
Facial palsy	0.92 (0.09—8.91)	.945	0.59 (0.11—1.29)	.385	7.46 (1.01—55.70)	.026
Diameter of T > 25 mm	1.81 (0.27—12.17)	.535	1.27 (0.64—2.23)	.869	1.69 (0.13—21.27)	.681
Advanced cT stage	1.86 (0.48—7.21)	.478	1.92 (0.28—7.37)	.371	0.97 (0.08—11.48)	.378
cN metastasis	2.00 (0.64—24.32)	.580	2.60 (0.40—6.68)	.878	0.94 (0.87—1.01)	.662
Adjuvant therapy	14.37 (1.42—142.35)	.007	1.30 (1.06—2.71)	.014	1.44 (1.02—2.07)	.004
PNI +	1.66 (1.05—2.76)	.008	1.21 (0.84—1.98)	.078	1.25 (0.92—1.70)	.071
LVI +	3.50 (0.43—28.13)	.221	0.75 (0.38—1.67)	.393	6.00 (0.31—116.60)	.190
R +	1.50 (0.67—3.34)	.087	1.52 (1.29—1.85)	.038	1.50 (0.67—3.34)	.087
Advanced pT stage	4.37 (0.46—42.08)	.174	1.95 (0.35—2.66)	.242	1.10 (0.96—1.27)	.219
pN metastasis	0.93 (0.84—1.02)	.206	–	1.000	0.69 (0.30—1.53)	.293

Abbreviations: CI, confidential interval; DFS = disease free survival; DSS = disease specific survival; LVI = lymph vascular invasion; N = lymph node; OS = overall survival; PNI = peri neural invasion; R = surgical margins; T = primary tumor

The wide confidence intervals observed for several variables reflect the limited number of events and should be interpreted with caution.

## Discussion

This systematic review and pooled analysis of 68 patients with BCAC represents one of the most comprehensive pooled patient-level analyses available to date in this rare salivary malignancy. Although BCAC has traditionally been described as a low-grade carcinoma with relatively favorable outcomes, the current findings confirm that the risk of recurrence and disease-related mortality is not negligible [4, 6, 17, 18, 33]. Nevertheless, the observed recurrence rate should not be interpreted exclusively as a direct surrogate of the intrinsic biological behavior of BCAC. Given the retrospective nature of the included literature and the long-time span covered, the pooled cohort likely reflects substantial heterogeneity in diagnostic workup, surgical management, pathological assessment, and indications for adjuvant therapy. However, the statistical analysis reported a 5-year DFS of 84.1%, as well as a 5-year DSS of 92.7%, and a 5-year OS of 88.3%. These results are in line with Seethala et al. [34], who reported a 5-year DSS of 90% and a 5-year OS of 87% in a multi-institutional series, and with Tian et al. [35], who found a 5-year DFS of 81.5% and a 5-year OS of 85.9%. Similarly, Guzzo et al. [36] documented a 5-year DFS of 83% and a DSS of 91% in 35 cases, while Ellis et al. [18] reported DSS rates above 90% in long-term follow-up. Moreover, Park et al. [17], in their single-institution cohort, observed comparable 5-year OS rates of 86%, reinforcing the consistency of these survival estimates across different populations.

The absence of adjuvant therapy was the most consistently significant factor in univariate analysis, significantly reducing LCD (p = 0.007), DFS (p = 0.007), DSS (p = 0.014), and OS (p = 0.004). This is consistent with Guzzo et al. [36] and Tian et al. [35], who reported improved DFS and DSS in patients receiving postoperative RT, especially in the presence of high-risk features such as PNI, R +, or advanced T stage. Similar evidence emerges from Terhaard et al. [37] and Garden et al. [38] in broader cohorts of salivary gland carcinomas, where adjuvant RT significantly improved 5-year DFS and OS. Ülkü et al. [6] additionally emphasized the role of postoperative RT in controlling local disease in high-grade BCAC cases, suggesting its potential benefit in preventing recurrences. These findings are consistent with ESMO guidelines, which recommend adjuvant RT in the presence of high-risk pathological features [39]. Adjuvant CHT was only sporadically reported in the dataset and is currently not recommended by established clinical guidelines in the adjuvant setting.

Nevertheless, the observed association between the absence of adjuvant therapy and worse outcomes should be interpreted with caution. The apparent mismatch between the frequency of high-risk pathological features and the relatively limited use of adjuvant RT likely reflects heterogeneity in clinical decision-making and, in some cases, suboptimal historical management across the included studies. Given the retrospective nature of case reports and case series, treatment indications were not standardized and were often incompletely reported. Therefore, the observed association should not be interpreted as a purely causal effect of adjuvant therapy on survival outcomes. Rather, it may also reflect that a subset of patients who did not receive adjuvant treatment—despite adverse pathological features—experienced worse outcomes. This finding is consistent with a potential indication bias and possible undertreatment effect. PNI was present in 40% of evaluable cases and significantly associated with worse LCD (p = 0.024) and DFS (p = 0.008), with a trend toward lower DSS (p = 0.078). Seethala et al. [34] found PNI-positive tumors had a 5-year DFS of 68% compared to 88% in PNI-negative cases. Ellis et al. [18] also identified PNI as an independent predictor of decreased DFS and DSS. Razali et al. [7] reported a case of BCAC with extensive PNI involving intracranial structures, highlighting the aggressive behavior linked to neural spread. This adverse impact is explained by the propensity of tumor cells to track along neural sheaths and invade regions beyond the surgical field, where both surgery and RT have limited effectiveness.

The presence of R + (27%) was associated with decreased DSS (p = 0.038) and borderline significance for LCD, DFS, and OS. In Seethala et al. [34], R + were linked to a 5-year DFS of only 55%, compared to > 85% in R0 cases. Ellis et al. [18] also reported significantly worse DSS and OS in patients with incomplete excision. Residual tumor cells persisting in the surgical bed act as a nidus for recurrence, which may explain the poor outcomes associated with R + [40]. Furthermore, Murty et al. [2] similarly emphasized that incomplete resection reduces the efficacy of adjuvant RT, as higher tumor burden at the time of irradiation decreases local control probability, ultimately leading to shorter survival rates.

Similarly, the rate of LVI (17%) showed a trend toward poorer LCD (p = 0.125), echoing Tian et al. [35], who both found LVI associated with reduced DFS. Advanced pathological T stage was associated with a tendency toward worse DFS (p = 0.174) and OS, consistent with Guzzo et al. [36], Terhaard et al. [37], and Mantsopoulos et al. [5]. Facial nerve palsy at presentation—found in 8% of our patients—was significantly associated with reduced OS (p = 0.026). Seethala et al. [34] reported OS rates of 55% in facial nerve–involved cases vs > 85% in uninvolved patients, supporting its value as a marker of aggressive disease. This finding is corroborated by Muller et al. [4] identifying facial nerve involvement as an indicator of advanced local disease with poorer prognosis. Although BCAC is typically considered a low-grade malignancy, high-grade transformation has been described in rare cases and is associated with more aggressive clinical behavior [35]. In the present analysis, tumor grading was inconsistently reported, and no cases of high-grade transformation were identified, limiting the ability to assess its prognostic impact. From a molecular perspective, BCAC has been associated with recurrent alterations in the CYLD gene, which plays a key role in tumor suppression pathways. CYLD mutations are also implicated in Brooke–Spiegler syndrome, a hereditary condition characterized by multiple adnexal tumors, including cylindromas, spiradenomas, and occasionally salivary gland neoplasms [41]. Rare cases of BCAC arising in patients with cutaneous cylindromatosis have been reported in the literature, suggesting a potential shared pathogenetic mechanism [42]. However, in the present analysis, molecular and syndromic data were largely unavailable, precluding any evaluation of these associations. Future studies integrating molecular profiling may provide further insights into the biological behavior and prognostic stratification of BCAC.

The observed discrepancy between clinical and pathological staging, with a higher proportion of advanced tumors at final histopathological assessment, suggests a potential underestimation of tumor extent at preoperative evaluation. This finding is consistent with the known limitations of imaging and cytology in distinguishing tumor infiltration in salivary gland neoplasms, and highlights the importance of surgical pathology for accurate staging. Additional findings indirectly support the presence of management heterogeneity within the pooled cohort. Preoperative FNAC was performed in only a minority of cases, clinical-pathological upstaging was frequent, and the extent of parotid surgery was variably reported, including limited resections in selected patients. These elements suggest that some historical cases may have undergone less comprehensive preoperative assessment or surgical treatment than would currently be recommended for a malignant parotid neoplasm.

The low rate of nodal metastasis and the presence of occasional occult nodal disease suggest that routine elective neck dissection may not be warranted in clinically node-negative patients, and that neck management should be individualized based on risk factors, although its role remains unclear in BCAC. Although uncommon, distant metastases—occurring in 7% of our cohort—were linked to poor prognosis. Most cases arose in patients with advanced T stage, PNI, or R + resections. Seethala et al. [34] observed a 5-year DSS drop from > 90% to < 50% in metastatic cases, while Guzzo et al. [36] and Tian et al. [35] documented similar impacts on OS.

Although the median follow-up duration in the present cohort was limited (36 months), previous studies have reported late recurrences and distant metastases occurring beyond the conventional 5-year period. Therefore, prolonged follow-up may be advisable, especially in patients with adverse pathological features. However, the relatively short follow-up in the current series may have led to an underestimation of late events, and these findings should be interpreted with caution. This recommendation is supported by the indolent biological behavior of BCAC, where disease progression can remain subclinical for extended periods. Seethala et al. [34] and Ellis et al. [18] reported cases of local or regional relapse occurring more than two years after apparently curative treatment, while Guzzo et al. [36] and Tian et al. [35] documented distant metastases manifesting even beyond the 5-year mark. Although the overall metastatic rate remains low (typically < 10%), such events often occur well after the completion of standard surveillance schedules. The current findings support the need for risk-adapted therapeutic strategies and prolonged surveillance in patients with parotid BCAC. In line with recent evidence on the management of rare head and neck tumors, optimal outcomes rely on individualized surgical planning within a multidisciplinary framework and on accurate pathological characterization to ensure appropriate risk stratification [41–44].

## Limitations and future perspectives

The present systematic review provides one of the most comprehensive patient-level analyses available on parotid BCAC; however, several limitations must be acknowledged. The rarity of this tumor results in small sample sizes and substantial heterogeneity across studies in terms of diagnostic workup, treatment strategies, and follow-up, which may limit the generalizability of the findings. Most included studies were retrospective case reports or small case series, often with incomplete and non-uniform reporting of key clinical and pathological variables, thereby reducing the robustness of subgroup analyses and introducing potential information bias. Importantly, the pooled cohort likely reflects variability—and in some cases possible inadequacy—of historical management. Differences in preoperative assessment, extent of surgery, and use of adjuvant radiotherapy, often inconsistently reported, may have influenced recurrence patterns and survival outcomes. Therefore, the observed recurrence rate may partly overestimate the intrinsic biological aggressiveness of BCAC and should be interpreted with caution. Furthermore, the lack of standardized indications for adjuvant therapy introduces a risk of indication bias, and the absence of multivariable analyses prevents adjustment for confounding factors. Additional limitations include the lack of molecular and genetic data, the inability to apply standardized classification systems such as ESGS, and the relatively short median follow-up, which may underestimate late recurrences. Wide confidence intervals in several analyses further reflect the limited number of events. Finally, publication bias cannot be excluded, as more aggressive or unusual cases are more likely to be reported.

Future research should focus on prospective, multicenter studies with standardized data collection and long-term follow-up. The development of international registries for rare salivary gland tumors and the integration of molecular profiling may improve risk stratification and guide more tailored therapeutic strategies.

## Conclusions

Parotid BCAC is generally associated with favorable outcomes; however, adverse pathological features may negatively impact prognosis. The present findings support the role of adjuvant RT in selected high-risk patients, in line with current clinical guidelines. Given the retrospective nature of the data and the presence of missing information, treatment recommendations should be interpreted with caution. Further prospective studies are needed to better define optimal management strategies.
